# Rehydrated ground corn silage as alternative to feedlot lambs: feeding behavior and performance

**DOI:** 10.1007/s11250-026-04942-w

**Published:** 2026-03-05

**Authors:** Leandro A. S. da Silva, Cláudia L. S. Lima, Gleidson G. P. de Carvalho, Henry D. R. Alba, Maria L. G. M. L. de Araújo, Cláudia H. da Cruz, Carlindo S. Rodrigues, Dalton H. Pereira, José E. de Freitas Júnior, Manuela S. L. Tosto, Douglas dos S. Pina

**Affiliations:** 1 Animal Science Department, Federal University of Bahia, Salvador, Bahia 40.170-110 Brazil; 2 Institute of Agricultural and Environmental Sciences, Federal University of Mato Grosso, Sinop, Mato Grosso 78.557-267 Brazil

**Keywords:** Ethology, High-concentrate diets, Ruminant nutrition, Sheep, Weight gain

## Abstract

This study aimed to investigate the effect of replacing ground corn with rehydrated ground corn silage (RGCS) in high-concentrate diets on the feeding behavior and performance of feedlot lambs. Forty, non-castrated, Santa Inês male lambs (approximately four months old with an average body weight of 21 ± 2.8 kg) were randomly assigned to a completely randomized design, with five dietary treatments, each including eight replications. The treatments consisted of replacing ground corn with RGCS at levels of 0, 250, 500, 750, and 1000 g/kg on a dry matter (DM) basis. The data collection period lasted 64 days. Replacement of ground corn with RGCS linearly decreased the intake of neutral detergent fiber (NDF) and its feeding and rumination efficiencies (*P* < 0.05). For each 1 g of RGCS included per kg of dietary DM, NDF intake, feeding efficiency of DM and rumination efficiency of NDF decreased by 0.093 g/day, 0.021 g/hour and 0.010 g/hour, respectively. Initial, final, total and average weight gains were not influenced by the diets (*P* > 0.05). Thus, it can be concluded that RGCS can replace up to 1000 g/kg of ground corn in high-concentrate diets without negatively affecting the feeding behavior or performance of feedlot lambs. This approach offers strategic flexibility in using corn grain - either in its dry form or as rehydrated and ensiled - based on production goals or market conditions in ruminant feeding systems. However, further research is needed, particularly concerning the economic aspects of its use.

## Introduction

Grains are the main energy source in concentrate diets for ruminants, with starch being the primary energy component (Su et al. 2025). Among these sources, corn grain is the most used ingredient in ruminant diets (Pereira et al. 2021; da Silva et al. 2022; Soares et al. 2024), often included at high levels (Rezende et al. 2018). In recent years, some diets have consisted exclusively of this ingredient (100% whole shelled corn), referred to as high-grain diets (Teixeira et al. 2025).

Beyond serving as a metabolizable energy (ME) source due to its high starch content - over 60% (Gomide et al. 2023) - corn is also palatable and highly digestible throughout the ruminant digestive system (Loy and Lundy 2018). The total tract digestibility of corn ranges from 91.2% to 98.9%, depending on the processing method and grain type, with ground corn averaging 93.5% (Poorkasegaran and Yansari 2014).

Rehydrated ground corn silage (RGCS) is produced by adding water to dry ground corn until a target moisture content of approximately 35% is achieved (Ferraretto et al. 2018; Mombach et al. 2019). The moistened grain is then stored in an anaerobic environment to initiate fermentation, during which microorganisms metabolize nutrients to produce short-chain organic acids (Roseira et al. 2023). This process enables effective feed preservation during periods of surplus production or limited storage capacity, while maintaining its composition to meet the dietary nutritional requirements of ruminants (da Silva et al. 2018).

The increase in starch degradability in high-concentrate diets is expected to enhance the production of volatile fatty acids, leading to changes in the ruminal environment. Such alterations may result in a reduction in dry matter intake (DMI)(da Silva et al. 2022). Therefore, evaluating feeding behavior is essential to establish the relationship between DMI and animal performance (Nielsen et al. 2016).

Assessments of changes in feeding behavior can provide relevant information about how animals consume feed and can be used as guide in feeding management to estimate feed intake and efficiency in increasing livestock productivity (Gougolis et al., 2010). Moreover, monitoring feeding, rumination, and idling activities is crucial to assess whether changes in the physical form, frequency, or site of digestion of the diet can influence animal productivity and health (Amorim et al. 2025). Monitoring these processes through the assessment of feeding behavior enables the development of more effective management strategies, because affects the animal’s performance.

The objective of the present study was to evaluate the effects of replacing ground corn with rehydrated ground corn silage in high-concentrate diets on the feeding behavior and performance of feedlot lambs. Thus, it was hypothesized that high-concentrate diets incorporating RGCS would enhance the productive performance of feedlot lambs without adversely affecting their feeding behavior.

## Materials and methods

### Experimental site and ethics approval

The experiment was carried out in the sheep feedlot of the Sheep Sector of the Experimental Farm of the Federal University of Bahia (UFBA). This farm is in the municipality of São Gonçalo dos Campos, state of Bahia, Brazil.

This study was conducted after being approved by the Committee of Ethics in the Use of Animals (CEUA) of the School of Veterinary Medicine and Animal Science of UFBA under protocol number 09/2020.

This is the second paper from one experiment that evaluated the effects of the substitution of ground corn by rehydrated ground corn silage (RGCS) in high-concentrate diets for feedlot lambs on feeding behavior and performance. The other study previously published by our group was carried out to determine how the RGCS affects the carcass characteristics and meat quality attributes of lambs (Silva et al. 2024).

### Animals and housing

Forty Santa Inês male lambs, non-castrated, approximately four months of age and with an average body weight of 21 ± 2.8 kg, were used. Each animal was individually housed in a 1.2 × 1.2 m pen located in a covered shed, equipped with a wooden slated floor. Pens were fitted with individual plastic feeders and water troughs. Throughout the experiment, lambs had *ad libitum* access to both feed and fresh water.

During the adaptation period, the lambs were identified, weighed (after a solid fast of 16 h), dewormed (albendazole, 2.5 mg/kg BW - Aldazol^®^, Vallée S.A., São Paulo, Brazil), vaccinated against rabies and clostridial infections (Gepec S.A., Belo Horizonte - MG, Brazil). Besides, animals were dewormed and treated for ectoparasites and submitted to supplementation with vitamins (1 ml per animal - ADE^®^ Vallée S.A., São Paulo, Brazil). Thus, during this period, the animals were adapted to the facilities, environment, management, and to experimental diets.

### Experimental design and treatments

The animals were randomly assigned to a completely randomized design, with five treatments, each including eight replications. The treatments consisted of replacing ground corn with RGCS at levels of 0, 250, 500, 750, and 1000 g/kg in the concentrate on a dry matter (DM) basis. Lambs were kept in 85 days of feedlot and allowed to adapt to the facilities, diets, and management for 21 days, which makes 64 days (experimental period).

The diets were formulated based on the National Research Council (NRC, 2007), to be isonitrogenous, with 160 g/kg of crude protein (CP) to meet the lambs’ requirements for an average daily gain (ADG) of 250 g/animal/day.

Animals were fed twice per day (09:00 and 16:00 h), divided equally into two meals, as a total mixed ration (TMR). Diets (DM basis) consisted of 300 g of roughage (corn silage) and 700 g of concentrate feed. The concentrate was comprised of corn germ, soybean meal, RGCS and/or ground corn, sodium bicarbonate, specific mineral salt for sheep, urea (as non-protein nitrogen source), ammonium sulfate, and limestone (Table 1).

**Table 1:** Raw material and chemical composition of the experimental diets with rehydrated ground corn silage as a substitute for ground corn in the concentrate (g/kg DM)

Item	RGCS level (g/kg DM)					RGCS	Ground corn
	0	250	500	750	1000		
Ingredients (g/kg DM)							
Corn silage	300	300	300	300	300	-	-
Ground corn	480	360	240	120	0	-	-
RGCS^1^	0	120	240	360	480	-	-
Soybean meal	130	130	130	130	130	-	-
Corn germ	50	50	50	50	50	-	-
Urea	10	10	10	10	10	-	-
Mineral²	14	14	14	14	14	-	-
Limestone	12	12	12	12	12	-	-
Sodium bicarbonate	6	6	6	6	6	-	-

Chemical composition (g/kg DM)							
Dry matter (*as-fed* basis)	745.2	708.0	670.8	633.5	596.3	578.0	888.3
Organic matter	943.5	943.4	943.3	943.2	943.1	985.4	986.2
Crude protein	165.5	166.8	168.1	169.4	170.7	94.6	83.8
Ether extract	45.1	45.3	45.5	45.7	45.9	41.0	39.3
Neutral detergent fiber	320.4	310.8	301.1	291.5	281.8	76.0	156.5
Non-fibrous carbohydrates	387.1	395.2	403.2	411.3	419.4	773.8	720.0
Total digestible nutrients	724.5	723.9	723.2	722.6	722.0	811.2	816.5

^1^Rehydrated ground corn silage; ^2^Assurance levels (per kilogram of active elements): 128 g Calcium; 44 g Phosphorus; 178 g Sodium; 12 g Sulfur; 5 g Magnesium; 107 mg Cobalt; 50 mg Copper; 50 mg Iodine; 750 mg Manganese; 12 mg Selenium; 3.7 g Zinc; 1.4 g Iron; 440 mg Fluorine

### The ensiling process

In the manner, to produce RGCS, dry corn grain was initially processed using a stationary forage chopper to achieve an average particle size of approximately 5.0 mm. The ground corn (70%) was then mixed with water (30%), thoroughly homogenized, and stored in 150 l plastic drum silos adapted with Bunsen-type valves. The mixture was packed to achieve a density of 700 kg/m³ on a fresh matter basis. Each silo was fitted with a removable lid and sealed with a metal ring to ensure anaerobic conditions.

Thus, similarly to our previous study, after 60 days of ensiling, the silage was directly used for diet formulation (Mombach et al. 2019). Consequently, the ensiling process used in the current study and management procedures with the animals were conducted in the same manner as the other study.

### Chemical composition of ingredients and diets

Samples of the feed ingredients, experimental diets, and refusals were collected and stored at -20 °C until analysis. Prior to analysis, the samples were thawed and dried in a forced-air oven at 55 °C for 72 h. Subsequently, they were ground using a Wiley-type mill (Marconi, MA-580, Piracicaba, São Paulo, Brazil) equipped with a 1-mm screen.

Samples were analyzed to determine dry matter (DM; Method 934.01), ash (MM; Method 942.05), crude protein (CP; Method 968.06), ether extract (EE; Method 920.39) contents according to Association of Official Analytical Chemists (AOAC 2005) procedures.

Neutral detergent fiber (NDF) and acid detergent fiber (ADF) were evaluated sequentially according to the method proposed by Van Soest et al. (1991). The non-fibrous carbohydrates (NFC) content was estimated following the method of Hall (2000), and total digestible nutrients (TDN) were determined according to da Cruz et al. (2021).

### Assessment of nutrient intakes and feeding behavior

Dry matter and NDF intakes were estimated by calculating the difference between the amounts of these nutrients in the feed offered and in the refusals. The daily feed allowance for each lamb was determined based on their prior feed intake, with refusals targeted at 10% to 20% (*as-fed* basis) of the total amount supplied to ensure *ad libitum* intake. Refusals were collected daily, weighed, and stored at -20 °C for subsequent chemical composition analysis.

Feeding behavior was observed individually and sequentially over a 24-hour period, with data recorded at 5-minute intervals, resulting in 288 observations per animal per day. Behavioral parameters including feeding, rumination, and idling times, and chewing activities were assessed at 28 and 52 days of trial, according to Johnson and Combs (1991).

Feeding behavior observations were conducted by nine trained observers, divided into three groups, each responsible for a consecutive three-hour observation period. Observers were positioned strategically to minimize interference with the animals’ natural behavior. During nighttime observations, the experimental area was artificially illuminated. Observations commenced at 09:00 h and continued for a full 24-hour cycle.

On the same day, the number of chews and the time spent ruminating each bolus were recorded using a digital stopwatch. For this evaluation, three cuds were monitored per animal during three specific time intervals: 10:00–12:00 h, 14:00–16:00 h, and 18:00–20:00 h. The total number of boluses ruminated per day was estimated according to the methodology proposed by Carvalho et al. (2017).

Feeding and rumination efficiencies for DM and NDF, total daily chewing time, and the combined duration of feeding and rumination were calculated according to the methodologies described by Bürger et al. (2000) and Polli et al. (1996).

### Growth performance

All lambs were weighed at the beginning, midpoint, and end of the experimental period (day 64) following a 12-hour period of solid-feed deprivation, during which water was provided *ad libitum*. The performance parameters evaluated included initial body weight, final body weight, total weight gain, ADG and feed conversion.

### Statistical analysis

Variables related to feeding behavior were analyzed using a completely randomized design as repeated measure arrangement. The effects of RGCS inclusion in the diet were assessed through orthogonal polynomial contrasts (linear and quadratic effects), using the MIXED procedure of the SAS statistical software (Version 9.4; SAS Institute Inc., Cary, NC, USA). The following mathematical model was used:

$$Y_{ijk} = \mu + RGCS_i + \varepsilon_{ij} + EVAL_k + (RGCS_{i} \times EVAL_k) +\varepsilon_{ijk},$$

Where: Y_*ij*_ = observed value in the portion that received the treatment *i* in repetition *j*; *µ* = general average, RGCS*i* = fixed effect of replacing ground corn for RGCS (*i* = 0, 250, 500, 750, and 1000 g/kg); and *Ɛ*_*ij*_ = random error to *RGCS*_*i*_ analysis with mean 0 and variance σ^2^, *EVAL*_*k*_ = number of feeding behavior evaluations (k = 2), *(RGCS*_*i*_ *x EVAL*_*k*_*) =* fixed effect of interaction between *RGCS* and *EVAL*,* Ɛ*_*ijk*_ random error to *EVAL*_*k*_ and *(RGCS*_*i*_ *x EVAL*_*k*_*)* analysis with mean 0 and variance σ^2^.

For the performance data, initial body weight mean (IBWM) was included as a covariate to account for baseline differences among animals. The analysis used the following statistical model:

$$Y_{ijk} = \mu + RGCS_i+\beta 1 \times (IBW_{ij}- IBWM)+ \varepsilon_{ij},$$

Where: *β*_*1*_ = common slope parameter; *IBW*_*ij*_ = IBW of animal *j* receiving level *i* of RGCS (covariate); *IBWM* = average value for covariate (initial body weight mean); *Y*_*ij*_; *µ; RGCS*_*i*_ and *Ɛ*_*ij*_ are the same described previously, in the first model.

Regression equations derived from orthogonal polynomial contrasts (linear and quadratic effects) were used to describe and interpret the effects of replacing ground corn with RCGS. A significance level of *P* < 0.05 was adopted for all statistical tests to control for Type I error.

## Results

### Feeding behavior

The times spent by lambs per activity on feeding, rumination and idling activities, and the percentages of their activities did not differ (*P* > 0.05) as a function of the substitution of ground corn for RGCS in the diet (Table 2). Thus, the lambs fed with RGCS at 1000 g/kg spent the same in the activities than those fed ground corn without hydration.

**Table 2:** Feeding behavior of feedlot lambs fed diets containing rehydrated ground corn silage as a replacement for ground corn

Item	RGCS level (g/kg DM)					SEM^1^	*P*-value^2^	
	0	250	500	750	1000		Linear	Quadratic
Time spent per activity (minutes)								
Feeding	212.32	177.19	184.38	202.20	179.37	13.96	0.363	0.506
Rumination	430.97	397.50	431.88	408.78	393.44	26.48	0.455	0.833
Idling	796.63	865.31	823.75	828.55	867.19	30.51	0.290	0.906
Activity percentages (%)								
Feeding	14.74	12.30	12.80	14.04	12.45	0.97	0.364	0.506
Rumination	29.92	27.60	29.99	28.38	27.32	1.88	0.455	0.833
Idling	55.32	60.09	57.20	57.53	60.22	2.12	0.291	0.906
Number of episodes								
Feeding	15.63	14.62	14.68	16.05	15.75	1.26	0.680	0.573
Rumination	25.36	25.93	25.12	24.22	23.25	1.08	0.095	0.439
Idling	38.03	38.37	37.06	37.56	36.18	1.50	0.352	0.777
Average time per episode (minutes)								
Feeding	14.04	12.45	12.56	12.99	11.87	0.79	0.139	0.670
Rumination	17.16	15.54	17.98	16.96	17.35	1.20	0.644	0.903
Idling	21.08	23.21	22.69	22.89	24.83	1.51	0.144	0.953
Chewing activities								
Number bolus/day	624.61	599.36	608.01	572.78	543.06	36.86	0.103	0.694
Seconds/bolus	64.04	62.32	70.10	65.78	67.92	3.72	0.342	0.753
Number/bolus	39.28	41.40	41.20	43.04	43.96	2.36	0.142	0.966
Number /day	40.470	36.590	42.910	3.,880	36.300	2.720	0.418	0.511
g DM/bolus	2.47	2.45	2.60	2.69	2.56	0.22	0.560	0.723

^1^Standard error of the mean; ^2^Probability for the effect of the corn ground substitution by RGCS, significant at *P* < 0.05

Regarding the number of episodes and the average time spent by lambs per episodes in feeding, rumination and idling were not influenced (*P* > 0.05) with the substitution of ground corn for RGCS in the diet (Table 2). Consequently, the replacement of ground corn with RGCS did not change the episodes of activities of the lambs, resulting in similar feeding behavior parameters.

In addition, the ground corn substitution by RGCS had no effect (*P* > 0.05) on the number of bolus/day, seconds per bolus, number/bolus, number/day and grams of DM/bolus of lambs (Table 2). Thus, rehydrated ground corn silage can completely replace ground corn in concentrate of high-grain diets with a 30:70 forage: concentrate ratio without affecting chewing activities in feedlot lambs.

The DM intakes did not differ (*P* > 0.05) as a function of the substitution of ground corn for RGCS in the diet (Table 3). Thus, rehydrated ground corn silage (RGCS) can replace 100% of the ground corn (GC) with diet concentrate without effect on major nutrient intakes by lambs. Nevertheless, NDF intake (*P* < 0.001), showed a linear decrease as ground corn was progressively replaced by RGCS. Specifically, for each 100 g of RGCS included per kg of dietary DM the NDF intake decreased by 9.30 g/day.

**Table 3:** Feeding and rumination efficiencies of feedlot lambs fed diets containing rehydrated ground corn silage as a replacement for ground corn

Item (g/hour)	RGCS level (g/kg DM)					SEM^4^	*P*-value^5^	
	0	250	500	750	1000		Linear	Quadratic
Nutrient intakes (g/day)								
Dry matter	1.536	1.394	1.518	1.467	1.354	60.86	0.142	0.607
Neutral detergent fiber^1^	455.0	369.9	399.7	383.9	331.7	18.60	< 0.001	0.775
Dry matter								
Feeding efficiency	450.1	489.0	497.6	445.8	483.5	36.54	0.841	0.653
Rumination efficiency	219.3	212.7	211.3	213.2	212.0	15.22	0.752	0.789
Neutral detergent fiber								
Feeding efficiency^2^	133.0	129.3	121.3	115.4	113.0	8.40	0.040	0.873
Rumination efficiency^3^	64.8	56.3	55.6	56.1	51.9	4.28	0.045	0.506

^1^Ŷ_INDF_ = 434.56–0.0931*×*RGCS (R^2^ = 66.46); ^2^Ŷ_NDFFE_ = 133.18–0.0216*×*RGCS (R^2^ = 97.21); ^3^Ŷ_NDFRE_ = 62.12–0.0104*×*RGCS (R^2^ = 74.72); ^4^Standard error of the mean; ^5^Probability for the effect of the corn ground substitution by RGCS, significant at *P* < 0.05

The substitution of ground corn by RGCS in high-concentrate diets did not alter (*P* > 0.05) the feeding and rumination efficiencies of DM by lambs (Table 3). Thus, ground corn substitution by RGCS up to 1000 g/kg DM in diets promotes similar DM feeding and rumination efficiencies in feedlot lambs.

On the other hand, feeding and rumination efficiencies of NDF decreased linearly with the substitution of ground corn for RGCS in the diet (*P* = 0.040 and *P* = 0.045)(Table 3). Thus, feeding efficiency of NDF and rumination efficiency of NDF decreased by 0.021 g/hour and 0.010 g/hour, respectively.

### Growth performance

Final body weights did not differ (*P* > 0.05) with the substitution of ground corn for RGCS in the diet (Table 4). Thus, lambs fed diets with RGCS up to 1000 g/kg DM had same body weights and developments in feedlot conditions as those fed ground corn.

**Table 4:** Performance of feedlot lambs fed diets containing rehydrated ground corn silage as a replacement for ground corn

Item	RGCS level (g/kg DM)					SEM^5^	*P*-value^6^	
	0	250	500	750	1000		Linear	Quadratic
IBW (kg)^1^	21.22	21.18	21.08	20.98	20.82	-	-	-
FBW (kg)^2^	37.95	37.48	38.16	37.81	37.15	1.10	0.720	0.733
TWG (kg)^3^	16.85	16.38	17.10	16.78	16.15	1.11	0.776	0.746
ADG (g/day)^4^	263.63	256.13	267.25	262.13	252.38	17.39	0.776	0.752

^1^Initial body weight; ^2^Final body weight; ^3^Total weight gain; ^4^Average daily gain; ^5^Standard error of the mean; ^6^Probability for the effect of the corn ground substitution by RGCS, significant at *P* < 0.05

Similarly, substitution of ground corn by RGCS in high-concentrate diets did not alter (*P* > 0.05) total weight and average daily gains in feedlot lambs (Table 4). Therefore, in the current study, diets containing (RGCS) substituting ground corn maintain the growth performance by lambs.

## Discussion

The absence of significant differences in DMI between diets in both our previous study (Silva et al. 2024) and the present one suggests that the fermentation of rehydrated ground corn during ensiling did not adversely affect the lambs’ feed intake capacity. In the current study, the complete replacement of ground corn with RGCS reduced the dietary DM content by 148.9 (ranging 745.2 to 596.3 g/kg of DM), reflecting the higher moisture content of the silage.

Concurrently, increasing the level of RGCS inclusion resulted in a reduction in NDF intake, which negatively impacted the feeding and rumination efficiencies of NDF. Lambs fed the ground corn-only diet exhibited higher averages for NDF feeding efficiency (133.18 g NDF/h) and rumination efficiency (62.12 g NDF/h) compared to those receiving the highest level of RGCS inclusion.

Although diet selection behavior was not assessed in this study, the observed reduction in NDF intake - coinciding with increased dietary moisture content - may suggest a diminished ability of the animals to selectively consume dietary components. This interpretation aligns with findings by Felton and DeVries (2010), who reported that increased moisture content can reduce sorting behavior in ruminants.

Despite differences in dietary composition, experimental diets did not affect the time lambs spent feeding, ruminating, or idling. According to Van Soest (1994), rumination time is primarily influenced by the fiber content and physical characteristics of the diet. In the current study, all treatments shared the same primary source of physically effective neutral detergent fiber (peNDF) - corn silage - allowing us to infer that both dry ground corn and RGCS exhibited similar effects on feeding behavior, despite differences in processing and preservation.

Interestingly, lambs spent the greatest proportion of their time idling, likely a result of the high concentrate inclusion in the diets. Mertens (1994) noticed that animals fed with high-energy, low-fiber diets tend to regulate intake based on energy needs, which may reduce feeding time and increase time spent idling. In this study, idling accounted for an average of 58.07% of the total observed behavioral activities.

Chewing activity is significantly influenced by diet composition, as it is closely linked to feeding and rumination time (Brandstetter et al. 2019). Diets containing RGCS had lower NDF levels and a reduced proportion of peNDF derived from non-forage components. Consequently, these diets likely provided less physical stimulation for chewing compared to diets with higher NDF and peNDF contents. According to Mendes et al. (2020), a reduction in NDF content and its effectiveness within the rumen environment can lead to decreased rumination and chewing times, which aligns with the findings observed in this study.

It is important to emphasize that the forage content remained constant regardless of the replacement of ground corn with RGCS, maintaining a consistent roughage-to-concentrate ratio. Therefore, differences in feeding behavior and intake parameters were likely influenced by the constitution of NFC, particularly starch, rather than variations in forage level. Ground corn served as the main NFC source, with differing effects attributable to the processing method - dry vs. rehydrated and ensiled. According to Poorkasegaran and Yansari (2014), variations in starch and NFC composition can affect feed intake, ruminal retention time, rumination duration, and overall chewing activity. Moreover, increased moisture associated with RGCS inclusion may have reduced the need for extensive mastication during eating by enhancing feed lubrication, thus facilitating easier swallowing (Havekes et al. 2020).

Although the overall fiber content differed among diets, the variation was not solely quantitative but also qualitative. The physical and chemical characteristics of fiber - such as particle size, density, fragility, moisture content, and digestibility - differed with the replacement of ground corn with RGCS and play a crucial role in influencing chewing activity, rumen mat formation, and rumen motility (Felton and DeVries 2010). These differences likely explain the behavioral patterns observed in feeding, rumination, and chewing activity in the present study.

Although it is speculated that the ensiling process may have enhanced digestibility through starch gelatinization and increased starch accessibility, no direct analyses of energy availability were conducted in this study beyond standard nutritional composition assessments.

Replacing ground corn with RGCS did not influence the growth performance of lambs in the present study. Consistent with these findings, da Silva et al. (2022) also reported no significant effects on performance parameters - including slaughter weight, hot and cold carcass weight when evaluating similar replacements. Likewise, Jacovaci et al. (2021) found no difference in ADG in feedlot young bulls fed diets in which dry corn grain was replaced by RGCS. However, in the last study there was a reduction in DMI, which the authors attributed to increased net energy content of the ensiled flint corn grain.

In the current study, the absence of an effect on DMI likely explains the lack of variation in animal performance. As highlighted by Silva et al. (2021), DMI is a primary determinant of productive outcomes in ruminants, accounting for up to 90% of performance variation, while diet digestibility contributes between 10% and 40% (Karimizadeh et al. 2017). Therefore, the similar performance across treatments in this study is justified by the consistency in DMI among the experimental groups.

## Conclusion

Based on our results, it can be concluded that rehydrated ground corn silage can replace up to 1000 g/kg of ground corn in high-concentrate diets without negatively affecting the feeding behavior or performance of Santa Inês feedlot lambs.

This finding is particularly significant in situations where ground corn cannot be stored in its dry form or when there is a need to purchase and store large quantities of corn during periods of lower prices. It provides a viable strategy as an alternative for times when corn prices are high or supply is limited, offering a flexible strategy for feed management in dynamic market conditions.

## Data Availability

None of the data was deposited in an official repository. This can be made available upon request to the corresponding author.

## Declarations

### Generative AI and AI-assisted technologies in the writing process

The authors did not use any artificial intelligence assisted technologies in the writing process.

## References

[CR2] Amorim MN, Costa DS, Harada ÉS, da Silva WP, Turco SHN (2025) Performance of electronic device and different visual observation intervals in assessing feeding behavior in sheep. Comput Electron Agric 231:110053. 10.1016/j.compag.2025.110053

[CR3] AOAC (2005) Official methods of analysis of the association of official analytical chemists, 16th edn. Association of Official Analytical Chemists Inc., Arlington, DC, USA

[CR4] Brandstetter V, Neubauer V, Humer E, Kröger I, Zebeli Q (2019) Chewing and drinking activity during transition period and lactation in dairy cows fed partial mixed rations. Animals 9:1088. 10.3390/ani9121088 31817555 PMC6941000

[CR5] Bürger PJ, Pereira JC, Queiroz ACD, Da Silva JFC, Valadares Filho SDC, Cecon PR, Casali ADP (2000) Ingestive behavior in Holstein calves fed diets with different concentrate levels. Rev Bras Zootec 29:236–242. 10.1590/S1516-35982000000100031

[CR6] Carvalho S, Frasson MF, Simões FSB, Bernardes GMC, Simões RR, Griebler L, Pellegrin ACRS, Menegon AM, Deponti LS, Severo MM, Mello VL (2017) Wet brewery residue in the finishing of feedlot lambs and its effects on the carcass characteristics and non-carcass components. Arq Bras Med Vet Zootec 69:742–750. 10.1590/1678-4162-8573

[CR7] da Cruz CH, Santos SA, de Carvalho GGP, Azevedo JAG, Detmann E, Valadares Filho SC, Mariz LDS, Pereira ES, Nicory IMC, Tosto MSL, Alba HDR (2021) Estimating digestible nutrients in diets for small ruminants fed with tropical forages. Livest Sci 249:104532. 10.1016/j.livsci.2021.104532

[CR8] da Silva MRH, Jobim CC, Neumann M, Osmari MP (2018) Corn grain processing improves chemical composition and fermentative profile of rehydrated silage. Acta Sci Anim Sci 40:1–6. 10.4025/actascianimsci.v40i1.42564

[CR9] da Silva MRH, Jobim CC, Neumann M, Osmari MP (2022) Substitution of dry corn grain by rehydrated and ensiled corn grain, finely or coarsely ground, on performance of young bulls finished in feedlot. Rev Bras Zootec 51:1–9. 10.37496/RBZ5120200160

[CR10] Felton CA, DeVries TJ (2010) Effect of water addition to a total mixed ration on feed temperature, feed intake, sorting behavior, and milk production of dairy cows. J Dairy Sci 93:2651–2660. 10.3168/jds.2009-3009 20494174

[CR11] Ferraretto LF, Silva Filho WI, Fernandes T, Kim DH, Sultana H (2018) Effect of ensiling time on fermentation profile and ruminal in vitro starch digestibility in rehydrated corn with or without varied concentrations of wet brewers grains. J Dairy Sci 101:4643–4649. 10.3168/jds.2017-14329 29519723

[CR12] Gomide DR, Pereira RAN, Silva RB, Carvalho JTR, Lara MAS, Pereira MN (2023) Effect of particle size of silage of Flint corn grain on dairy cows fed tropical pasture: performance, intake, ruminal fermentation, and digestibility. Animals 13:1932. 10.3390/ani13121932 37370442 PMC10295517

[CR13] Gougoulis DA, Kyriazakis I, Fthenakis GC (2010) Diagnostic significance of behaviour changes of sheep: A selected review. Small Rumin Res 92:52–56. 10.1016/j.smallrumres.2010.04.018

[CR14] Hall MB (2000) Neutral detergent-soluble carbohydrates: nutritional relevance and analysis. University of Florida, Gainesville, Florida, USA

[CR15] Havekes CD, Duffield TF, Carpenter AJ, DeVries TJ (2020) Moisture content of high-straw dry cow diets affects intake, health, and performance of transition dairy cows. J Dairy Sci 103:1500–1515. 10.3168/jds.2019-17557 31837778

[CR16] Jacovaci FA, Salvo PAR, Jobim CC, Daniel JLP (2021) Effect of ensiling on the feeding value of Flint corn grain for feedlot beef cattle: A meta-analysis. Rev Bras Zootec 50:e20200111. 10.37496/rbz5020200111

[CR17] Johnson TR, Combs DK (1991) Effects of prepartum diet, inert rumen bulk, and dietary polyethylene Glicol on dry matter intake of lactating dairy cows. J Dairy Sci 74:933–944. 10.3168/jds.S0022-0302(91)78243-X 1649204

[CR19] Karimizadeh E, Chaji M, Mohammadabadi T (2017) Effects of physical form of diet on nutrient digestibility, rumen fermentation, rumination, growth performance and protozoa population of finishing lambs. Anim Nutr 3:139–144. 10.1016/j.aninu.2017.01.004 29767114 PMC5941103

[CR20] Loy DD, Lundy EL (2018) Nutritional properties and feeding value of corn and its coproducts. In: Serna-Saldivar SO (ed) Corn, 3rd edN. AACC International Press Inc., St. Paul pp 633–659. 10.1016/B978-0-12-811971-6.00023-1

[CR21] Mendes JAC, Parente MOM, Parente HN, Zanine AM, Ferreira DJ, Moreira Filho MA, Da Cunha IAL, Ladim AV, Da Rocha KS (2020) Performance, ingestive behavior and cost of production of finishing lambs fed non-forage diets. Biol Rhythm Res 51:460–470. 10.1080/09291016.2018.1535540

[CR22] Mertens DR (1994) Regulation of forage intake. In: Fahey Jr. GC (ed) Forage quality, evaluation, and utilization. American Society of Agronomy, Inc. Crop Science Society of America, Inc. Soil Science Society of America, Inc. pp. 450–493. 10.2134/1994.foragequality.c11

[CR23] Mombach MA, Pereira DH, Pina DS, Bolson DC, Pedreira BC (2019) Silage of rehydrated corn grain. Arq Bras Med Vet Zootec 71:959–966. 10.1590/1678-4162-9676

[CR25] National Research Council – NRC (2007) Nutrient requirements of small ruminants: sheep, goats, cervids, and new world camelids. National Academies, Washington, DC, USA

[CR24] Nielsen BL, De Jong IC, De Vries TJ (2016) The use of feeding behavior in the assessment of animal welfare. In: Phillips CJC (ed) Nutrition and the welfare of farm animals. Springer, New York, NY, USA, pp 59–84. 10.1007/978-3-319-27356-3_4.

[CR27] Pereira DM, Santos EM, Oliveira JS, Santos FNS, Lopes RC, Santos MAC, Corrêa YR, Justino ES, Leite GM, Gomes PGB, Cruz GFL, Torres Júnior PC (2021) Effect of cactus Pear as a moistening additive in the production of rehydrated corn grain silage. J Agric Sci 159:731–742. 10.1017/S002185962100099X

[CR28] Polli VA, Restle J, Senna DB, Almeida RS (1996) Rumination of bovine and Bubaline steers in feedlot regimen. Rev Bras Zootec 25:987–993. 10.1590/S0103-84781995000100024

[CR29] Poorkasegaran S, Yansari AT (2014) Effects of different sources of carbohydrates on intake, digestibility, chewing, and performance of Holstein dairy cows. J Anim Sci Biotechnol 5:1–11. 10.1186/2049-1891-5-6 24410961 PMC3912492

[CR30] Rezende PLP, Restle J, Bilego UO, Fernandes JJR, Missio RL, Menezes RG, Guimarães TP (2018) Digestibility and feeding behavior of cattle fed soybean hulls to replace corn in high concentrate diets. Semin Cienc Agrar 39:363–372. 10.5433/1679-0359.2018v39n1p363

[CR32] Roseira JPS, Pereira OG, da Silveira TC, da Silva VP, Alves WS, Agarussi MCN, Ribeiro KG (2023) Effects of exogenous protease addition on fermentation and nutritive value of rehydrated corn and sorghum grains silages. Sci Rep 13:7302. 10.1038/s41598-023-34595-w 37147458 PMC10162983

[CR33] Silva BDC, Pacheco MVC, Godoi LA, de Souza GAP, Trópia NV, Pucetti P, Silva FAS, Menezes ACB, Rennó LN, Paulino MF, Schoomaker JP, Valadares Filho SDC (2021) Feed intake, nutrient digestibility, and selected rumen parameters in feedlot bulls fed diets with different feed additives. PlosOne 16:e0259414. 10.1371/journal.pone.0259414 PMC8562795 34727141

[CR34] Silva LA, Lima CL, Pina DDS, Alba HDR, de Araújo MLGML, Cirne LG, Azevêdo JAG, Rodrigues CS, Borges LM, Chaves MLO, de Carvalho GGP (2024) Carcass traits and meat quality of lambs fed with rehydrated ground corn silage. Small Rumin Res 231:107193. 10.1016/j.smallrumres.2023.107193

[CR35] Soares RL, de Oliveira JS, Santos EM, de Araújo GGL, Santos FNS, Gomes PGB, Justino ES, Pereira DM, Cavalcanti HS, Leite GM, Torres Júnior PC, Santos MAC, Viana NB (2024) Corn grain rehydration methods: water vs. cactus Pear in the diet for feedlot lambs. Small Rumin Res 230:107151. 10.1016/j.smallrumres.2023.107151

[CR36] Su X, Zhang L, Sun Y, Wu Y, Ren J, Wu S, Lei X, Zhang J, Wang D, Ren H, Yao J (2025) Net energy of grains for dairy goats differed with processing methods and grain types. J Anim Sci Biotechnol 16:3. 10.1186/s40104-024-01136-y 39755637 PMC11700460

[CR37] Teixeira PD, Rodrigues AC, Casagrande DR, Batista ED, Júnior JMO, Santos AC, Paulinho PVR, Ladeira MM (2025) Total nutrient digestibility and small intestine starch digestion in Nellore and Nellore × Angus steers fed whole shelled corn-based diet with or without roughage. Livest Sci 296:105699. 10.1016/j.livsci.2025.105699

[CR39] Van Soest PJ (1994) Nutritional ecology of the ruminant, 2nd edn. Cornell, Ithaca

[CR38] Van Soest PV, Robertson JB, Lewis BA (1991) Methods for dietary fiber, neutral detergent fiber, and nonstarch polysaccharides in relation to animal nutrition. J Dairy Sci 74:3583–3597. 10.3168/jds.S0022-0302(91)78551-2 1660498

